# Impact of high-speed handpiece noise-induced dental anxiety on heart rate: analyzing experienced and non-experienced patients - a comparative study

**DOI:** 10.1186/s12903-024-04017-y

**Published:** 2024-02-23

**Authors:** Raahim Salman Abdul Ghaffar, Mahnoor Sheikh, Muneeza Kidwai, Anas Sanaullah, Mousab Salman, Anum Ilyas, Naseer Ahmed, Abhishek Lal

**Affiliations:** 1 Aga Khan University, Karachi, Pakistan; 2 Altamash Institute of Dental Medicine, Karachi, Pakistan; 3 Azerbaijan Medical University, Baku, Azerbaijan; 4 Department of Prosthodontics, Altamash Institute of Dental Medicine, Karachi, Pakistan; 5 Department of Medicine, The Aga Khan University, Karachi, Pakistan

**Keywords:** Hand piece, Heart rate, Dental anxiety

## Abstract

**Background:**

Dental anxiety is very much common among the patients and could be due to different factors like the behavior of the dentist, past experiences, Needle phobia, or word of mouth from other patients. According to recent studies, a strong association between sound and anxiety has been found, so this observational study has been conducted to find out the link between the activation of anxiety with the sound of a handpiece between experienced patients, who have already gone through the dental treatments and non-experienced patients.

**Methods:**

Total of 297 participants were part of this study. These participants were divided into 2 groups according to the experienced and non-experienced dental patients. The researcher first filled out the CORAH Dental Anxiety Scale (DAS) form to mark the anxiety level of the patients, and then noted the readings of the heart rate in 3 intervals which were before during, and after the treatment with the pulse oximeter. Later the data was analysed using the SPSS independent t-test.

**Results:**

Results show that patients in group 1 who have gone through the dental treatment before were less anxious and had a lesser effect on their heart rate than the patient who were having the treatment for the first time who were in group 2. Another interesting factor was noticed that in both the groups female were found to be more anxious than male participants. Participants with younger age were found to be more anxious than older age patient in both groups

**Conclusions:**

The sound of the handpiece can provoke anxiety in the patient, affecting the heart and increasing the heart rate. Participants who were experienced were found to be less anxious than the participants who were inexperienced.

## Introduction

Dental anxiety is one of the big issues which is so common in the population that, it is now considered normal. Dental anxiety (DA) is defined as that it is a state of apprehension, coupled with a sense of losing control, which is linked to a feeling that something dreadful is going to happen concerning dental treatment [1]. It is very much harmful to the person not only during the treatment but also in their daily life is being affected. The study showed that a person suffering from dental anxiety also faces the physiological impact which is the fright response, cognitive impact which includes memory loss, wrong beliefs, and negative feelings, social impact, and health impact which can be sleep deprivation or oral health-related [2]. So, it is of paramount importance to identify such patients to alleviate their dental anxieties which may have an impact on their daily lives.

Perception of dental anxieties varies among patients as every person has a different state of mind [3]. Previous traumatic dental experiences trigger dental anxieties among patients. Moreover, personal traits and negative dental experiences of friends and family further worsen the already fearful individual [4].

Sound or noise can also be another factor, which can also be responsible for behavioral changes in humans, as it can generate feelings of fear, surprise, and anxiety among individuals [5]. In normal Dental practice, dental equipment such as handpiece, suction tube, and scaler tips used during the different procedures generate noises. In many hospitals, it is suggested that there should be a “Department of Sound” which should control and maintain the environment of the hospitals and practice so that it would be beneficial for the patient to reduce Heart rate, breathing rate, anxiety, and pain [6]. The sound in general hospital practice is measured as 66 to 70 decibels (dB) is enough to provoke anxiety and other behavior impacts [7]. One study evaluated, that the sound pressure produced by the high-speed handpiece is 87.3 dB, and another study it is mentioned 102 dB [8], which is more than enough to bring the state of anxiety in patients, which can be detected through the pulse oximeter because it has been stated in the previous studies that there is the strong association between the psychological and physiological effects as anxiety is considered as psychological stress and it affects the physiological condition of the body i.e. Heart rate [9]. As heart rate increases during the state of anxiety and stress [10]. The level of anxiety is also being studied in the previous studies, it is also variable among individuals with different ages, genders, and treatment types [3]. Keeping all previous results of studies in the center this study is conducted to evaluate the anxiety level among the population, by the noise of high-speed Handpiece, among the patients who have already experienced the treatment and patients who are going through the treatment for the first time, through the heart rate measured by the pulse oximeter.

## Material and method

### Study design

The observational study was conducted from September 2022 to January 2023, at Altamash Institute of Dental Medicine, Department of Operative Dentistry and Endodontics. The ethical approval for this study was granted by the ethical review committee of Altamash Institute of Dental Medicine (AIDM/ERC/07/2023/01). After enrolment of the participant, the data collection was started. The data was collected in three sections which included First, the questionnaire made by the researchers for the demographic details. Second, the CORAH Dental Anxiety Scale (DAS) measuring tool was asked to the patients to fill before the start of the treatment to measure their general dental anxiety score. The DAS measuring scale helps to indicate the emotional reaction to the Dental Visit. The reliability was good with an internal consistency of 0.86 and the test–retest was 0.82 [11]. This scale relies upon a scoring system. The scores range from 4 to 20, and the patient’s level of anxiety is quantified as follows: a total score of 4 indicates “no fear”, a score between 5 and 8 corresponds to “low fear”, a score between 9 and 14 indicates “moderate fear”, and a score between 15 and 20 corresponds to “high fear” [12]. The questionnaire was in the English language and patients were asked to fill out this form before the placing of oximeter Finally, readings of the heart rates were recorded by the researcher during the intervals of the procedure.

The demographic data includes Age, Gender, Occupation, and experienced and non-experience patients, which means that if the patient ever had dental treatment before, specifically Root canal treatment and teeth fillings, if yes, they were entered into the category of experienced patients and if no, they were mentioned into the non-experienced patients The patients were explained the nature of the study and its potential outcomes. After verbal and written informed consent for voluntary participation, patients were enrolled in the study.

For the final heart rate readings collection, the patient was seated at the corner-most dental chair of the department to minimize the other sound effects, this was standardized for the all the patients which were part of this study. The measuring of anxiety level, which is the main objective of the study was done by using the Pulse oximeter, which was placed on the right index finger of the patient, after management of the seating position of the dentist and patient. The researcher recorded the readings on the pulse oximeter 3 times during the procedure, Firstly, it was recorded when the position was set and before the start of the treatment. The second reading was recorded, without removing the oximeter after the first reading, when the dentist held the High-speed Handpiece and pressed the paddle outside the mouth for about 5 s. A handpiece was placed near the patient so that they could hear the sound. The final reading was taken following the removal of cavities and the crown-cutting process, marking the cessation of handpiece usage, which is the mandatory procedure for both filling and root canal treatments. The handpiece that was used during the procedure was the Apple Dental-LED handpiece, which creates the sound as mentioned on the website is ≤ 65db [13].

### Sampling technique and sample size

The participant of the study was selected through the non-probability convenience sampling technique. The eligibility criteria for the involvement of the participant were;

- Patients belonging to the American Society of Anaesthesiology (ASA) Class 1,
- Patient undergoing root canal treatment or filling of the tooth
- Patient aged between 18 and 50 years old
- Patient does not have any medical condition or drugs which alter the heart rate or anxiety control pills.

This medical history was asked from the patients during the diagnosis when patient visited to the hospital, and excluded from the study. Patients with co-morbidities, undergoing other dental procedures, and those who declined to provide their consent were not part of the study.

To calculate the sample size, we used the ClinCalc Sample Size Calculator, keeping the confidence interval at 95, the power of the test at 80%, and the margin of error at 0.05. The sample size was calculated to be 282 [14].

### Data management and analysis

The data collected was analyzed using SPSS version 25.0 (Statistical Package for Social Sciences, IBM). Mean and standard deviations were calculated for the descriptive variables. The heart rates of experienced and non-experienced patients were compared using independent t-tests. A *p*-value of ≤ 0.05 was considered to be statistically significant.

## Results

This cross-sectional study had a total of 297 participants with 151 (51.5%) participants in Group 1 (Experienced) and 146 (48.5%) participants in Group 2 (Non-experienced). The gender distribution of males and females in each group is as follows: 78 and 73 in Group 1 Experienced and 73 and 73 in Group 2 Non-experienced, as presented in Table 1 in Appendix.

The mean score of the CORAH Dental Anxiety Scale (DAS) measuring tool, for both groups, results in: 7.63 ± 2.92 among which experienced patients have a mean score of 7.00 ± 3.03 and non-experienced scored 8.00 ± 2.66. According to the criteria, about 62% of the participants were mildly anxious in the experienced group while 50% were mildly anxious in the non-experienced group. About 4% of the group reached a severe level of anxiety in the non-experienced group. However, none of the patients suffered from severe anxiety in the experienced group. The rest were categorized as moderate which is about 35% and high at about 3% in experienced participants while moderate about 41% and high at 2% in non-experienced participants.

The mean heart rates of participants in Group 1 and Group 2 were as follows 80.17 ± 9.36 and 87.70 ± 7.72. Amongst the two groups, Group 1 participants reported a maximum reduction in heart rate from before to after treatment, as compared to Group 2. Participants that belonged to group 1 reported suffering less anxiety as denoted by their heart rates before, during, and after the treatment in comparison to group 2 participants. About the comparison of heart rate between the two groups using an independent t-test, a statistically significant difference was found before treatment (*p* = 0.0020), during treatment (*p* = 0.001), and after treatment (*p* = 0.001), as presented in Table 2 in Appendix.

Regarding the levels of gender anxiety, females in group 1 reported slightly higher heart rates as compared to males. Similarly, in group 2, females reported higher heart rates as compared to males. Regarding the level of anxiety for age, younger individuals in both groups reported higher levels of anxiety as compared to older individuals.

## Discussion

Dental anxiety is the emotional state or fear from dental stimuli and dental procedures preceding an encounter, as they cause significant distress to the patient. Dental anxiety is an oral health problem that leads to fewer dental visits and a higher prevalence of oral diseases [15]. Dental anxiety is one of the major issues that lead to the avoidance of patients from getting routine dental checkups and proper dental treatment. It affects 5–7% of our population that rarely visits a dental professional [16]. There are a few factors associated with dental anxiety being prevalent in certain patients such as cynicism towards dentists, unsatisfactory oral hygiene, less frequent dental visits, avoidance of routine dental check-ups being female, and lower income [17] Noise is one of the factors that can cause severe anxiety and stress causing a psychological impact on the patients [18]. Noise may stimulate fear which may be associated as a leading factor of dental anxiety [19]. Our study found that there was a significant difference in the heart rates of the experienced and non-experienced patients, among which females had a higher heart rate than males. Although few males have greater heart rates than females, overall females’ heart rates were higher than males, which can be justified by the previous study that males are more prone to have higher heart rates and heart disease than females [20].

The goal of our study was to investigate the association of dental anxiety with the noise produced from a high-speed handpiece in experienced patients (those who have visited a dental professional before) and non-experienced patients (those who have not visited a dental professional before our survey) and association of different factors with it. The evaluation method of our study to measure the anxiety level of the participant patients was through the heart rate of the patients measured by pulse oximeter and through Corah’s Dental Anxiety Scale (DAS). Our study had 297 participants who visited the oral dentistry department for root canal treatments and fillings. 151 patients out of the 297 patients were experienced and 146 were non-experienced.

The observations made in our study revealed that patients who have already been through dental treatment before with a CORAH DAS mean score of 7.00 ± 3.03, are less anxious than patients who are having treatment for the first time, having a CORAH (DAS) score of 8.00 ± 2.66. This result is also supported by another study which showed that the first dental visit or the previous traumatic experience does affect the anxiety level of the patient, which means that patients are more anxious when they are visiting the clinic for the first time [21], that is also supported by this study results as both the group experienced and non-experience face increased in heart rate during the noise of handpiece but after that experience group has remarkable depression in heart rate as compared to the non-experienced patient. Even after proper counseling and instructions non-experienced patient seemed nervous about what this machine going to do to their teeth and what they would feel after that. This post-procedure feeling can be part of future research which can give more stronger standing for this study. In contrast, we have also found in other results of the studies that second patients having previous experience with dental treatment can be more anxious due to their personal bad experience or trauma which they had in their childhood or age before [22].

Moreover, our study concluded that females were more anxiety-prone as compared to males. This could be because as mentioned in the previous studies, females are more open to their feelings hence they express their anxiety more openly as compared to males. Moreover, females have a lower threshold of pain and tend to panic more easily as compared to male patients [23–25]. However, these results might not be supported by the other studies which indicated that gender has no significant relationship with dental anxiety [26]. The reason could be that this study measured the anxiety related to dental injections and injections that can scare the patients irrespective of gender. Our study is related to sound and it specifies that sound anxiety can be gender specific. Another study justified our results by mentioning that different factors like observing the needle, the sound of the handpiece, and infrequent visits can also induce anxiety in females. Most importantly, it highlighted that the position of the chair can also contribute to muscle tension, eventually leading to anxiety [27]. Nonetheless, it has been noticed that females show more positive attitudes towards their oral health, as they visit dental clinics more frequently, demonstrating better oral health behaviour, and having more oral health literacy compared to their male counterparts [28]. One thing to mention is important According to the standard ISO protocols 7785:199716, the sound of the handpiece should be below or equal to 65 dB [29] but in our study, we have seen that using of handpiece whose voice is ≤ 65 dB still creates anxiety for the patient.

Age and education are other factors that revealed a positive correlation. The level of anxiety is indirectly proportional to the level of educational awareness. If the patient is educated and properly counseled i.e. that giving patients clear and helpful advice about their health before the start of the treatment, anxiety levels diminish [30]. Onset of anxiety related to dental treatment usually takes place in childhood as proven by the study showing 50% of the prevalent value. About 21% in adolescence and 27% in adulthood [31]. The same trend can be noticed in this study which stated higher anxiety levels in younger participants compared to the elder participants.

It is advisable to make the dental office environment and the patient’s dental experience as stress-free as possible through various methods [32]. The most common stimuli that trigger dental anxiety are injection, the sight and sound of a handpiece, and the pain of the treatment in question. It is important to recognize the reason behind patients’ anxiety and deal with it accordingly. The ideal way is to use a method called systemic desensitization in which the patient is exposed to the means of anxiety in question for familiarity and to remove the element of surprise in the patients [33].

## Strengths and limitations of the study

One of the most important strengths of this study is it consists of a good sample size and researchers have gained multiple readings of the heart at different steps of the procedures. The limitation of this study is that this study was performed at the Outpatient Department (OPD) of Operative and Endodontics in Altamash Institute of Dental Medicine, where minimization of the other sound effects was nearly a challenge.

So, it can be the future recommendation for the reader and future researchers to perform such studies in a private and isolated clinical setup, as it reduced the bias and give the results near accuracy.

## Conclusion

It has been concluded that the sound of the High-speed handpiece induces anxiety among patients. With a higher ratio of heart rate for non-experienced patients than the experienced patient. Gender has a significant impact as female patients were more anxious than male patients also patients of younger age were highly anxious than the older age.

## Figures and Tables

**Fig. 1:**
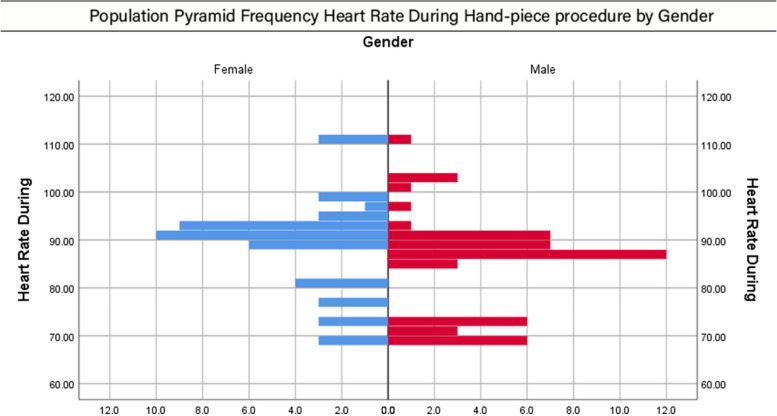
Comparison of Heart Rate During Treatment between Males and Females

**Fig. 2:**
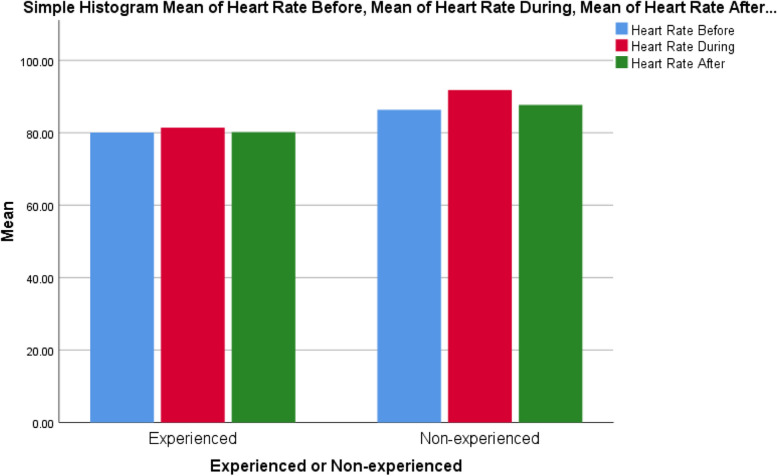
Mean Values of Heart Rates Before, During and After Treatment between Experienced and Non-Experienced Groups.

## Data Availability

The datasets used and analysed during the current study are available from the corresponding author on reasonable request.

## Appendix

**Table 1:** Sociodemographic characteristics of the participants (*n* = 99)

Groups	Variables	Mean and Standard Deviation
Experienced	Age	40.41 ± 11.16
	Gender (Males and Females)	78 and 73
Non-experienced	Age	31.58 ± 12.03
	Gender (Males and Females)	73 and 73

**Table 2:** Comparison of heart rates between experienced and non-experienced before, during, and after treatment

		**Mean**	**Std**	**t**	**df**	**Mean Difference**	**Std. Error Difference**	**95% Confidence Interval of the Difference**		***p*****-value**
								**Lower**	**Upper**	
Heart Rate Before	Experienced	80.05	10.41	-3.2	97	-6.27	1.94	-10.14	-2.40	0.002
	Non-experienced	86.33	8.85	-3.2	96.02	-6.27	1.93	-10.12	-2.42	
Heart Rate During	Experienced	81.41	9.50	-5.7	97	-10.37	1.81	-13.97	-6.78	0.001
	Non-experienced	91.79	8.43	-5.7	96.67	-10.37	1.80	-13.96	-6.79	
Heart Rate After	Experienced	80.17	9.36	-4.3	97	-7.53	1.73	-10.96	-4.09	0.001
	Non-experienced	87.70	7.72	-4.3	95.37	-7.53	1.72	-10.94	-4.11	
